# The Efficacy of Transcanal Endoscopic Ear Surgery in Children Compared to Adults

**DOI:** 10.3390/children12040519

**Published:** 2025-04-17

**Authors:** Wan-Hsuan Sun, Chia-Ho Chen, Chao-Yin Kuo, Sheng-Yao Cheng, Tzu-Chin Huang

**Affiliations:** 1 Department of Otolaryngology, Tri-Service General Hospital, National Defense Medical Center, Taipei 11490, Taiwan; steve88085@gmail.com (W.-H.S.); AllenQ0417@gmail.com (C.-H.C.); chefsketchup@hotmail.com (C.-Y.K.); gjcheng5032@gmail.com (S.-Y.C.); 2 Department of Otolaryngology, Cathay General Hospital, Taipei 10630, Taiwan

**Keywords:** transcanal endoscopic ear surgery, pediatric, chronic otitis media, cholesteatoma

## Abstract

**Background/Objectives**: Using transcanal endoscopic ear surgery to manage middle ear disease in children remains a controversial topic. The aim of this study was to compare the efficacy of transcanal endoscopic ear surgery between children and adults. **Methods**: The aim of this study was to compare the efficacy of transcanal endoscopic ear surgery between children and adults. **Results**: We observed no significant difference between pediatric and adult patients with regard to the rates of surgical success, postoperative hearing gain, and postoperative complications in all surgical procedures. As for ventilation tube insertion, the pediatric patients had shorter surgical times compared to adult patients. With respect to tympanoplasty, the pediatric group had a longer surgical time than adults did. **Conclusions**: Our study showed that transcanal endoscopic ear surgery can be successfully applied to manage various middle ear diseases in children.

## 1. Introduction

Microscopic ear surgery (MES) is a well-developed traditional surgical procedure for treating middle ear disease. However, the microscope provides only a straight-line view through the ear canal, and blind spots and hidden recesses can easily be missed using this transcanal approach. This problem is especially common in the management of cholesteatomas and can increase the risk of a residual cholesteatoma due to remnants of the matrix in the ear [1]. An otologist often must adopt the retroauricular transmastoid approach to overcome the limited surgical view of the microscope, which inevitably leaves an obvious surgical wound behind the ear.

Recently, transcanal endoscopic ear surgery (TEES) has been proven to be an effective alternative to traditional MES for treating middle ear disease in adults due to its wider and more flexible surgical view, better mastoid function preservation, smaller surgical wound, shorter surgical time, and shorter hospital stay [1,2,3,4,5]. However, the feasibility of TEES in pediatric patients is still debatable because children’s external auditory canals (EACs) are narrower, shorter, and curvier than those of adults [1]. In this study, the surgical outcomes and surgical time (duration of surgery) were compared between adult and pediatric patients who underwent TEES, and we also discussed the feasibility of TEES in children.

## 2. Materials and Methods

### 2.1. Patients

This study was conducted from January 2014 to August 2016 in Cathay General Hospital, Taiwan. We enrolled a total of 31 ears of 20 pediatric patients (aged 3–18 years) and 102 ears of 86 adult patients (aged 24–78 years) with chronic otitis media (COM) who underwent TEES in this study. Among the subjects, 13 children (23 ears) and 23 adults (34 ears) had chronic otitis media with effusion (OME), 4 children (5 ears) and 46 adults (51 ears) had COM without cholesteatomas, and 3 children (3 ears) and 17 adults (17 ears) had cholesteatomas. OME and COM diagnoses may reflect underlying eustachian tube dysfunction or atelectasis, though these were not separately classified as primary indications. (Table 1).

### 2.2. Patient Evaluation

Preoperative and postoperative evaluations included otoscopic examination and audiometric assessment. Furthermore, for patients with cholesteatomas, preoperative imaging was performed using temporal bone high-resolution computed tomography (HRCT). We recorded the surgical outcome, postoperative complications, preoperative and postoperative audiometric results, and surgical time in detail to carry out the comparison.

#### 2.2.1. VTI for Adults

In adult patients undergoing VTI, endoscopic examination typically revealed a retracted or immobile tympanic membrane with visible middle ear effusion, consistent with OME. No additional abnormalities, such as ossicular chain disruption or cholesteatoma, were noted in the VTI subgroup.

#### 2.2.2. Preoperative Otoscopic Assessments Documented Perforation Characteristics

Based on available records, perforations in both groups were typically central or marginal, involving the pars tensa, with no significant bias toward specific quadrants (e.g., anterior vs. posterior) between groups. Regarding size, our dataset includes perforation percentages for some patients (e.g., ranging from 20 to 90% in adults and 30 to 90% in children for tympanoplasty cases), with mean perforation sizes of 55% in children and 51.2% in adults (*p* = 0.73, as noted in the supplementary data). These findings suggest comparable perforation sizes between groups, though variability existed within each cohort. Preoperative otoscopic examinations assessed perforation location (primarily central or marginal) and size (mean 55% in children, 51.2% in adults), with no significant group differences (*p* = 0.73).

#### 2.2.3. Preoperative High-Resolution Computed Tomography (HRCT) Findings for All Cholesteatoma Cases

While specific HRCT findings were not detailed in the original manuscript, our supplementary data categorize cholesteatoma extent as (1) confined to the attic (Extent 1), (2) involving the attic and antrum (Extent 2), or (3) extending to the mastoid proper (Extent 3). In children, cholesteatomas were distributed as 2 attic-confined (Extent 1) and 1 with antrum involvement (Extent 2); in adults, 7 were attic-confined, 7 involved the antrum, and 3 extended to the mastoid (supplementary data). Ossicular erosion was observed in cases requiring ossiculoplasty (2/3 pediatric, 10/17 adult ears), and bone erosion (e.g., scutum or canal wall) was noted in advanced cases, particularly Extent 3 (Table 2).

### 2.3. Surgical Procedures

The TEES was performed using a 0- or 30-degree wide-angle endoscope with a 3 mm diameter and 14 cm length (HOPKINS^®^ Telescopes, KARL STORZ SE & Co. KG–Tuttlingen, Dr.-Karl-Storz-Straße 34, 78532 Tuttlingen, Germany). All operations were conducted by the same surgeon (senior author). Three different TEES procedures were adopted in order to manage different diseases.

#### 2.3.1. Ventilation Tube Insertion (VTI) for OME

Ventilation tube insertion was performed under general anesthesia in pediatric patients and under local anesthesia in adults. The procedure included myringotomy on the anterior-inferior quadrant of the tympanic membrane, followed by drainage of the middle ear fluid and interposition of the ventilation tube through the eardrum. Different ventilation tubes (Sheehy-Type Collar Buttons, Medtronic Inc., 800 53rd Ave NE, Minneapolis, MN 55421, USA) with diameters of 1.02 and 1.27 mm were selected for pediatric and adult patients, respectively. Irrigation with ofloxacin (3 mg/mL) (Tarivid; Daiichi Sankyo, 3-5-1, Nihonbashi-honcho, Chuo-ku, Tokyo 103-8426, Japan) was performed following ventilation tube stabilization.

#### 2.3.2. Tympanoplasty for COM Without Cholesteatoma

Tympanoplasty was also performed under general anesthesia for children and under regional local anesthesia for adults (2% lidocaine hydrochloride and 1:50,000 epinephrine) with injection on the skin of the tragus and ear canal. The graft material (including the perichondrium and cartilage) was harvested from the meatal surface of the tragus, and the wound was sutured with Vicryl 5-0 (Coated VICRYL^®^ polyglactin 910 Suture, Ethicon, 1000 US Highway 202 S Raritan, NJ 08869, USA). After denuding the perforated edge of the eardrum, a transmeatal incision (straight upward) was made to elevate the tympanomeatal flap. All the inflammatory tissue in the tympanic cavity was then completely removed, and the tympanic cavity was cleaned using saline irrigation. Furthermore, the ossicular chain was checked, and ossiculoplasty was simultaneously performed if such was deemed necessary. The skin of the anterior canal and the annulus were elevated, and Surgifoam (Spongostan Gelatin Hemostatic Sponge, Ethicon, 1000 US Highway 202 S Raritan, NJ 08869, USA) soaked with ofloxacin (3 mg/mL) (Tarivid; Daiichi Sankyo, 3-5-1, Nihonbashi-honcho, Chuo-ku, Tokyo 103-8426, Japan) was placed in the middle ear cavity. The perichondrium graft was introduced underneath the perforated eardrum; then, the tympanomeatal flap was repositioned. The ear canal was then packed with Surgifoam soaked with ofloxacin.

#### 2.3.3. TEES for Cholesteatoma

**Figure 1:**
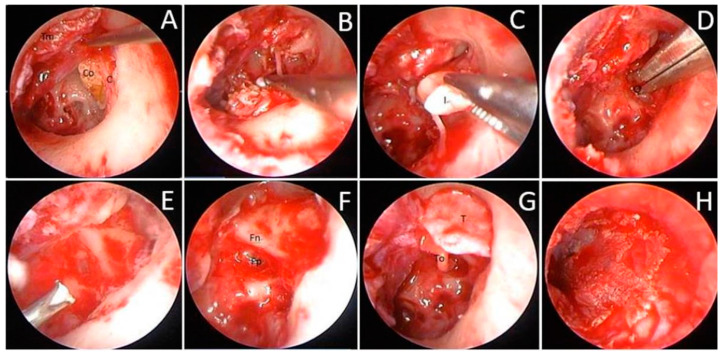
Congenital cholesteatoma removal using TEES in a 5-year-old girl: (**A**) After elevating the tympanomeatal flap, the cholesteatoma behind the chorda tympani was visible. Co = cholesteatoma; C = corda tympani nerve; Tm = tympanomeatal flap; (**B**) After drilling the scutum, the surgeon carefully removed the cholesteatoma; (**C**) The incus was destroyed, and the stapes could not be identified during surgery. Therefore, the destroyed incus was removed. I = destroyed incus; (**D**) The residual cholesteatoma at the sinus tympanum and the matrix at the oval window were removed. O = oval window; (**E**) Water irrigation of the middle ear; (**F**) Identification of the location of the footplate and facial nerve. Fp = footplate; Fn = facial nerve; (**G**) The scutum was reconstructed using tragus cartilage. Meanwhile, the ossicular chain was restored using total ossicular replacement prosthesis. T = tragus cartilage; To = total ossicular replacement prosthesis (TORP); (**H**) Replacement of the tympanomeatal flap.

HRCT findings guided surgical planning, identifying cholesteatoma extent (attic-confined: 2/3 pediatric, 7/17 adult ears; antrum involvement: 1/3 pediatric, 7/17 adult ears; mastoid extension: 0/3 pediatric, 3/17 adult ears), ossicular erosion (2/3 pediatric, 10/17 adult ears), and bone erosion in advanced cases. Attic-confined cholesteatomas allowed closed cavity reconstruction, while mastoid involvement necessitated open cavity procedures to ensure complete removal. For this condition, the graft tissue, including the perichondrium and cartilage, was also harvested from the meatal surface of the tragus. Then, an elongated transmeatal incision was made to elevate the tympanomeatal flap. Next, the scutum was drilled to expose the attic area. The dissection was extended along the cholesteatoma to the bottom of the matrix until the cholesteatoma could be completely removed. If the ossicular chain was involved and destroyed by the cholesteatoma, ossiculoplasty was performed simultaneously as necessary. The reconstruction procedure was slightly modified according to the extent of the dissection in each case. In patients who had limited cholesteatomas confined within the attic, the tympanic cavity and scutum were reconstructed using the composite cartilage and perichondrium (close cavity procedure) (Figure 1) [6]. In patients who had advanced disease involving the mastoid cavity proper, we reconstructed only the tympanic cavity and left the mastoid antrum as an open cavity in the ear canal (open cavity procedure).

For cholesteatomas, atticotomy was performed to access the attic, with further dissection based on extent. Attic-confined cases underwent closed cavity reconstruction (cartilage/perichondrium). Cases with mastoid involvement required extended dissection, leaving an open cavity (mastoid antrum exposed, not canal wall-down mastoidectomy).

Postoperative follow-up (18–48 months) included otoscopic and audiometric assessments to monitor for cholesteatoma recurrence, defined as new keratin accumulation or mass. Second-look procedures or MRI were not routinely performed unless clinically indicated, which was not required in this cohort.

The performance of all of the above TEES procedures left no surgical wound outside the ear canal. After the operations, patients were regularly followed up in the outpatient clinic for 18 to 48 months. For patients who underwent tympanoplasty, successful surgery was defined as complete healing of the tympanic membrane; for those with cholesteatomas, it was defined as complete removal of the cholesteatoma without any residual or recurrent disease during the postoperative follow-up.

## 3. Theory/Calculation

### 3.1. Theory

Comparison of TEES in children and adults

To determine the feasibility of TEES in children, these two groups were compared with respect to (1) surgical outcome, which included the rates of surgical success, hearing restoration (postoperative hearing gain), and postoperative complications, and (2) surgical time (duration of surgery).

### 3.2. Calculation

#### 3.2.1. Patients

Patient characteristics are summarized in Table 1; 53% of the subjects were male. The mean ages of the pediatric and adult patients were 8.4 and 49.5 years, respectively. The most frequent procedure among the pediatric patients was VTI (74%), followed by tympanoplasty (16%) and mastoidectomy (10%); among adult patients, the majority underwent VTI (33%), followed by tympanoplasty (50%) and mastoidectomy (17%) (Table 1).

#### 3.2.2. Surgical Outcomes

No complications were noted following the surgery in either of our study groups. Regardless of the surgical procedure, all patients demonstrated improvement in the air-bone gap after the surgery. The results showed no significant difference between the pediatric and adult groups with respect to the rates of surgical success and postoperative hearing gain; the same was observed whether VTI, tympanoplasty, or cholesteatoma TEES management was performed. Both VTI and mastoidectomy had 100% success rates in each group. All patients recovered well, with the patients having either satisfactory ventilation tube function after VTI or no residual or recurrent cholesteatoma after mastoidectomy. We found no significant difference between children and adults regarding the success rate of tympanoplasty, although the rate was lower in the pediatric patients (80% in children vs. 96% in adults, *p* = 0.253). For continuous outcomes (e.g., hearing gain, surgical time), mean differences with 95% confidence intervals and Cohen’s d effect sizes were calculated to assess clinical significance alongside *p*-values (Table 3 and Table 4).

#### 3.2.3. Surgical Time

The management of cholesteatoma required the longest surgical time (average of all cases, 156 min.); this was not surprising due to it being a more sophisticated procedure. However, no difference was observed between the pediatric and adult groups with regard to the surgical time (pediatric group, 158.3 min; adult group, 155.6 min; *p* = 0.897).

The average surgical time for tympanoplasty was 89.6 min in all cases and was longer in the pediatric patients than in the adult patients (pediatric group, 118 min; adult group, 86.6 min; *p* < 0.05).

The surgical time for VTI in children was shorter than in adults (pediatric group, 11.7 min; adult group, 19.1 min; *p* < 0.05) (Table 5).

## 4. Results

### 4.1. Surgical Outcomes

We observed no significant difference between these two groups with regard to rates of surgical success, postoperative complications, and postoperative hearing gain when VTI, tympanoplasty, or TEES management of cholesteatoma was performed. All patients achieved improved air-bone gap, and none experienced residual or recurrent disease during the 18- to 48-month postoperative follow-up after the management of cholesteatoma. Therefore, we believe that TEES may also have a satisfactory surgical outcome in children as well as in adults (Table 2).

### 4.2. Surgical Time

The average surgical time for VTI was longer in adults than in children because the procedure was performed under local anesthesia in adults; the injection of the local anesthesia in the ear canal may account for the longer surgical time. The longer tympanoplasty duration in children may partly reflect challenges with larger or marginal perforations, which require more meticulous repair, compounded by narrower ear canals.

Our study results revealed that the surgical time for tympanoplasty was significantly longer in the pediatric patients than in the adult patients. Children’s ear canals are narrower than those of adults at the orifice and isthmus, which increases the difficulty of endoscopic transcanal manipulation; these factors may have also increased the surgical time [7]. However, the average surgical time in the pediatric group still fell within satisfactory limits.

In the treatment of cholesteatoma, no statistically significant difference was found between these two groups with respect to surgical time because the TEES management of cholesteatoma is quite a sophisticated and complicated procedure in both children and adults; therefore, it generally has a longer surgical time regardless of the group (Table 3).

In pediatric patients, narrow ear canals occasionally complicate visualization and instrument manipulation, necessitating angled endoscopes or smaller instruments. These challenges, while manageable, likely contributed to longer operative times compared to adults.

## 5. Discussion

### 5.1. The Feasibility of TEES in Pediatric Patients

In recent years, an increasing number of studies have proven the benefits of TEES in managing middle ear diseases. For the management of cholesteatoma, better visualization of the residual cholesteatoma in the hidden space can be provided by an endoscope [1,5,7,8,9,10,11,12,13,14,15,16,17,18,19,20,21]. Such an endoscope can also provide a high-resolution image in order to clearly identify the tympanic segment of the facial nerve. The facial recess can be easily explored using an endoscope with an angled view without curetting or drilling the surrounding structure around the facial nerve [4,11]. Furthermore, with transcanal endoscope-assisted middle ear surgery, most of the healthy structures and mastoid air cells, as well as the mucosal gas exchange and mastoid buffer, can be preserved, which are crucial for restoring middle ear function and reducing post-surgical morbidity [1,2,5,8].

Study have also suggested that the time needed for endoscopic ear surgery is shorter than that required for traditional microscopic ear surgery. Using the endoscopic transcanal approach facilitates faster access to the pathologic lesion directly through the ear canal without drilling the mastoid cavity, which may thus significantly reduce the surgical time [4]. However, in our study, we did not include a comparison group undergoing microscopic ear surgery and therefore cannot draw direct conclusions about differences in surgical time between these two techniques. Our findings are limited to comparisons of surgical time and outcomes between pediatric and adult patients undergoing TEES.

Most of the previous studies were conducted in adult patients, while a few were conducted in pediatric patients. Studies on the applicability of TEES in pediatric patients are lacking, and thus the efficacy and feasibility of TEES in children remain controversial. The external auditory canal (EAC) in children is shorter and narrower than that of adults, and whether this may limit the application of TEES in pediatric patients with middle ear disease is still under investigation. In a study by Sun et al. on the anatomical applicability of TEES in children, the authors provided anatomical evidence and suggested that TEES can be a safe and effective alternative in the treatment of middle ear disease in children with appropriate endoscopes and instruments [7]. Some authors have already reported the successful clinical application of endoscopes in middle ear surgery in children [5,12,13].

Our study showed that pediatric patients can have as good surgical outcomes (including the rates of surgical success, postoperative complications, and postoperative hearing gain) as adult patients, regardless of the surgical procedure. The surgical time required for TEES management of cholesteatoma was similar in both the pediatric and the adult patients. Although the surgical time of tympanoplasty was longer in children, it still fell within an acceptable range.

Perforation size and location likely affect surgical complexity and outcomes, but their consistent impact across groups appears mitigated by the enhanced visualization of TEES.

Future studies with extended follow-up (e.g., 5–10 years) are recommended to confirm the durability of TEES outcomes in both age groups.

### 5.2. Study Limitations

We have only begun to adopt TEES for the management of middle ear disease in recent years. The number of patients in our study was still limited, especially in the pediatric group. Furthermore, the follow-up period was not long enough (18 to 48 months) to obtain long-term results. We think that additional studies with more patients and long-term follow-up may be required to generate more definitive conclusions. The lack of detailed, standardized documentation of perforation characteristics (e.g., precise location and size) may limit the precision of outcome comparisons, as these factors could influence surgical complexity and success rates.

The small sample sizes, particularly for pediatric tympanoplasty (5 ears) and cholesteatoma surgery (3 ears) compared to adults (51 and 17 ears, respectively), reduced statistical power, potentially masking subtle outcome differences. These sizes reflect the lower prevalence of these conditions in children but reduce statistical power, potentially masking subtle differences in outcomes.

This limits the generalizability of our findings, especially for less common procedures in children. Additionally, the 18–48-month follow-up period may be insufficient to detect late cholesteatoma recurrences, which can occur beyond this timeframe.

The lack of detailed documentation on intraoperative difficulties (e.g., bleeding or restricted access due to narrow canals) limits our ability to fully characterize pediatric-specific challenges, which may influence surgical complexity.

Although the difference in tympanoplasty success rates between children (80%) and adults (96%) was not statistically significant (*p* = 0.253), the lower success rate in children suggests a potential clinical difference that may be obscured by the small pediatric sample size (5 ears vs. 51 in adults). This limits our ability to definitively conclude equivalence in outcomes across age groups.

## 6. Conclusions

We believe that TEES is an effective approach for treating middle ear disease in both adults and children, achieving high success rates overall (e.g., 100% for VTI and cholesteatoma surgery). However, tympanoplasty success was lower in children (80%) compared to adults (96%), indicating that outcomes may not be equivalent across age groups, potentially due to anatomical challenges like narrower ear canals or differences in disease pathology, even in complicated cholesteatoma cases. However, further studies involving a larger number of patients are warranted to confirm our hypothesis.

## Figures and Tables

**Table 1:** Patient Characteristics.

Variable	VTI	Tympanoplasty	Cholesteatoma Surgery
	Children	Adults	Children
Indication	OME	OME	COM without Cholesteatoma
Ears (number)	23	34	5
Mean age (years)	7.6	50.1	11.2
Age range (years)	3–18	24–78	6–15
Male sex (%)	60.9%	82.4%	20.0%

Cholesteatoma Surgery: Includes atticotomy with closed or open cavity reconstruction based on cholesteatoma extent. This ensures consistency and clarity.

**Table 2:** Cholesteatoma Characteristics and Surgical Approach.

Variable	Children (3 Years)	Adults (17 Years)
Location/Extent (HRCT)		
Attic-confined (Extent 1)	2 (66.7%)	7 (41.2%)
Attic + Antrum (Extent 2)	1 (33.3%)	7 (41.2%)
Mastoid Extension (Extent 3)	0 (0%)	3 (17.6%)
Ossicular Erosion	2 (66.7%)	10 (58.8%)
Bone Erosion	0 (0%)	3 (17.6%)
Surgical Approach		
Closed Cavity (Attic Reconstruction)	2 (66.7%)	7 (41.2%)
Open Cavity (Mastoid Exposure)	1 (33.3%)	10 (58.8%)

**Table 3:** Comparison of surgical outcome of TEES between pediatric and adult patients.

Variable	VTI			Tympanoplasty			Mastoidectomy		
	Children (23)	Adults (34)	*p*	Children (5)	Adults (51)	*p*	Children (3)	Adults (17)	*p*
Hearing gain (dB)	18.3	17.8	0.932	7.4	10.3	0.386	5.2	11.3	0.337
Preoperative air-bone gap	23.5	24.3	0.783	21	24.8	0.321	28.5	29.7	0.866
Postoperative air-bone gap	4.8	9.9	0.051	13.7	14.5	0.842	23.3	18.5	0.517
Success rate (%)	100	100	-	80	96	0.253	100	100	-
Postoperative complication rate (%)	0	0	--	0	0	--	0	0	--

**Table 4:** (Excerpt). Surgical Outcomes.

Procedure	Outcome	Children	Adults	Mean Difference (95% CI)	Effect Size (Cohen’s d)	*p*-Value
Tympanoplasty	Hearing Gain (dB)	7.4 ± 11.1	10.3 ± 6.5	−2.9 (−9.7, 3.9)	0.31	0.386
Cholesteatoma	Success Rate (%)	100%	100%	-	-	-

**Table 5:** Comparison of surgical time of TEES between pediatric and adult patients.

Variable	VTI			Tympanoplasty			Mastoidectomy		
	Children (23)	Adults (34)	*p*	Children (5)	Adults (51)	*p*	Children (3)	Adults (17)	*p*
**Surgical time (min)**	11.7	19.1	<0.05	118	86.6	<0.05	158.3	155.6	0.897

## Data Availability

The original contributions presented in the study are included in the article; further inquiries can be directed to the corresponding author.

## Supplementary Materials

The following supporting information can be downloaded at: https://www.mdpi.com/article/10.3390/children12040519/s1.
